# Dopamine production in the brain is associated with caste-specific morphology and behavior in an artificial intermediate honey bee caste

**DOI:** 10.1371/journal.pone.0244140

**Published:** 2020-12-17

**Authors:** Ken Sasaki, Mariko Harada

**Affiliations:** 1 Department of Bioresource Science, Tamagawa University, Machida, Tokyo, Japan; 2 Honeybee Science Research Center, Tamagawa University, Machida, Tokyo, Japan; University of Alberta, CANADA

## Abstract

Caste polymorphism in eusocial insects is based on morphological plasticity and linked to physiological and behavioral characteristics. To test the possibility that dopamine production in the brain is associated with the caste-specific morphology and behavior in female honey bees, an intermediate caste was produced via artificial rearing using different amounts of diet, before quantifying the dopamine levels and conducting behavioral tests. In field colonies, individual traits such as mandibular shape, number of ovarioles, diameter of spermatheca, and dopamine levels in the brain differed significantly between workers and queens. Females given 1.5 times the amount of artificial diet that control worker receives during the larval stage in the laboratory had characteristics intermediate between castes. The dopamine levels in the brain were positively correlated with the mandibular shape indexes, number of ovarioles, and spermatheca diameter among artificially reared females. The dopamine levels were significantly higher in females with mandibular notches than those without. In fighting experiments with the intermediate caste females, the winners had significantly higher dopamine levels in the brain than the losers. Brain levels of tyrosine were positively correlated with those of catecholamines but not phenolamines, thereby suggesting a strong metabolic relationship between tyrosine and dopamine. Thus, the caste-specific characteristics of the honey bee are potentially continuous in the same manner as those in primitively eusocial species. Dopamine production in the brain is associated with the continuous caste-specific morphology, as well as being linked to the amount of tyrosine taken from food, and it supports the aggressive behavior of queen-type females.

## Introduction

Caste polymorphism in eusocial insects is based on phenotypic plasticity to allow specialization in particular tasks by different colony members. The phenotypes are usually divided discontinuously into different morphological and behavioral types in advanced eusocial species, whereas those in primitively eusocial species are continuous with overlapping morphology between workers and queens, including body size [1–4]. Under natural conditions, individuals with intermediate characteristics between castes rarely occur in advanced eusocial species [2,3].

In advanced eusocial Hymenoptera, including honey bees, the female caste is determined by nutritional states in the early larval stage. In female honey bee larvae, feeding on royal jelly during the first three larval instars leads to a queen phenotype [3,5]. Sugars and royalactin in royal jelly are the key factors responsible for the queen phenotype, whereby they mediate signaling by insulin, target of rapamycin, epidermal growth factor, and juvenile hormone [5–9]. Nutrition of female larvae can be manipulated by nurse workers in the nest, such that discontinuous phenotypes of the castes in advanced eusocial Hymenoptera might be caused by the fine manipulation of feeding by workers [10].

Dopamine is a biogenic amine that functions as a neurotransmitter, neuromodulator, and neurohormone in the central and peripheral nervous systems of insects [11–13]. In the honey bee brain, dopamine is a physiological characteristic that differs between castes. Queens possess large amounts of dopamine and its metabolites in the brain and hemolymph [14,15]. The caste-specific differences in the dopamine levels in the brain are generated during pupal development via the differential expression of genes encoding enzymes involved in dopamine biosynthesis [16]. The large amount of dopamine in virgin queens may contribute to queen-specific behavior, including their high aggressiveness against rival virgin queens [17], high locomotor activity [18], and high flight activity for mating [19]. Reproductive workers also have higher dopamine levels in the brain than normal workers [20–22]. The higher dopamine levels in reproductive workers may be associated with egg-laying behavior and ovarian activation in honey bees [23–25]. The effects of dopamine on reproduction, as well as correlations between dopamine and ovarian activation have been reported in reproductive females in bumble bees [26,27], a paper wasp [28,29], and ants [30–32]. Thus, dopamine is a key substance that mediates caste-specific behavior and ovarian activation in primitive and advanced eusocial Hymenoptera.

In contrast to the caste-specific external morphology and some internal morphological features determined during the late larval and prepupal stage in advanced eusocial insects [33–35], the neuroendocrine characteristics and behavior are determined during the late pupal or early adult stage [16,36,37]. Therefore, it is hypothesized that the neuroendocrine state changes based on caste-specific morphology and modulates behavior in adult females in advanced eusocial insects. A pertinent question is whether females subjected to a continuous caste-specific morphology will modify their dopamine production if continuous morphology can be produced artificially in advanced eusocial insects. Addressing this question might help to understand the evolution of caste differentiation implications in the brain from primitive to advanced eusocial species.

The artificial rearing of female honey bee larvae is a powerful technique for investigating the mechanisms that might underlie caste differentiation [38–42]. This technique has been used for determining the nutritional factors in the diet that affect caste determination and for comparing the morphological characteristics between caste types [7,9,43–45]. It is possible to produce an artificial intermediate caste of female honey bees using this artificial rearing method. Therefore, to test the possibility that the brain dopamine levels are associated with continuous morphology and the modulation of behavior, we produced an artificial intermediate caste in female honey bees, before conducting behavioral experiments and subsequently quantifying their dopamine levels in the brain.

## Materials and methods

### Artificial rearing of larvae

Female larvae were obtained one day after egg hatching from worker comb cells in queenright honey bee (*Apis mellifera*) colonies. The larvae were each transferred into a plastic queen cup with an artificial diet (S1 Table). The queen cups were placed in a 6 × 8-well plate, which allowed their removal if the larvae died. The procedure employed for artificially rearing larvae, especially the composition and amount of artificial diet, was as described by [42]. The composition and amount of artificial diet was set for each larval age (S1 Table). Under control fed condition for raising workers, the larvae were fed the artificial diet in the range of 10 to 50 μL depending on the age (“control”). We also prepared larvae fed 1.5 times the amount of control diet in the range of 15 to 75 μL (“1.5×fed”). The well plates with larvae were kept in a plastic box with 95% humidity in an incubator maintained at 32°C. When the larvae became prepupae, the remaining diet seen sometimes under 1.5×fed condition was removed from the cup and a piece of filter paper was added. During the pupal stage, the well plate was kept at approximately 80% humidity and 32°C. After adult emergence, 5–10 control females were kept in a cage (8.5 cm × 4 cm × 1.7 cm) with 15% sucrose solution for three days. A 1.5×fed female with 10 workers taken from the nest were kept in the cage with 15% sucrose solution for three days, and used in the experiments.

To compare the artificially and naturally reared individuals, worker pupae developing on a regular worker comb frame in field colonies were transferred into an incubator kept at 32°C. The newly emerged workers were kept in the cage with 15% sucrose solution for three days. To obtain emerged queens, larvae aged 1–2 days were grafted into each queen cup and introduced into a queenless area separated by a queen excluder in a queenright colony [46]. During the pupal stage for queens, a queen cell containing a queen pupa was transferred into an incubator kept at 32°C. A newly emerged queen and 10 workers were kept in a cage with 15% sucrose solution for three days, and used in the experiments.

Three-day-old adult females reared under artificial and natural condition were euthanized with liquid nitrogen. The heads were stored in liquid nitrogen for measurements of dopamine levels and mandibular morphology, and the abdomens were stored in a freezer kept at -80°C for measurements of internal morphology until use.

### Evaluation of morphological characteristics

**Fig 1:**
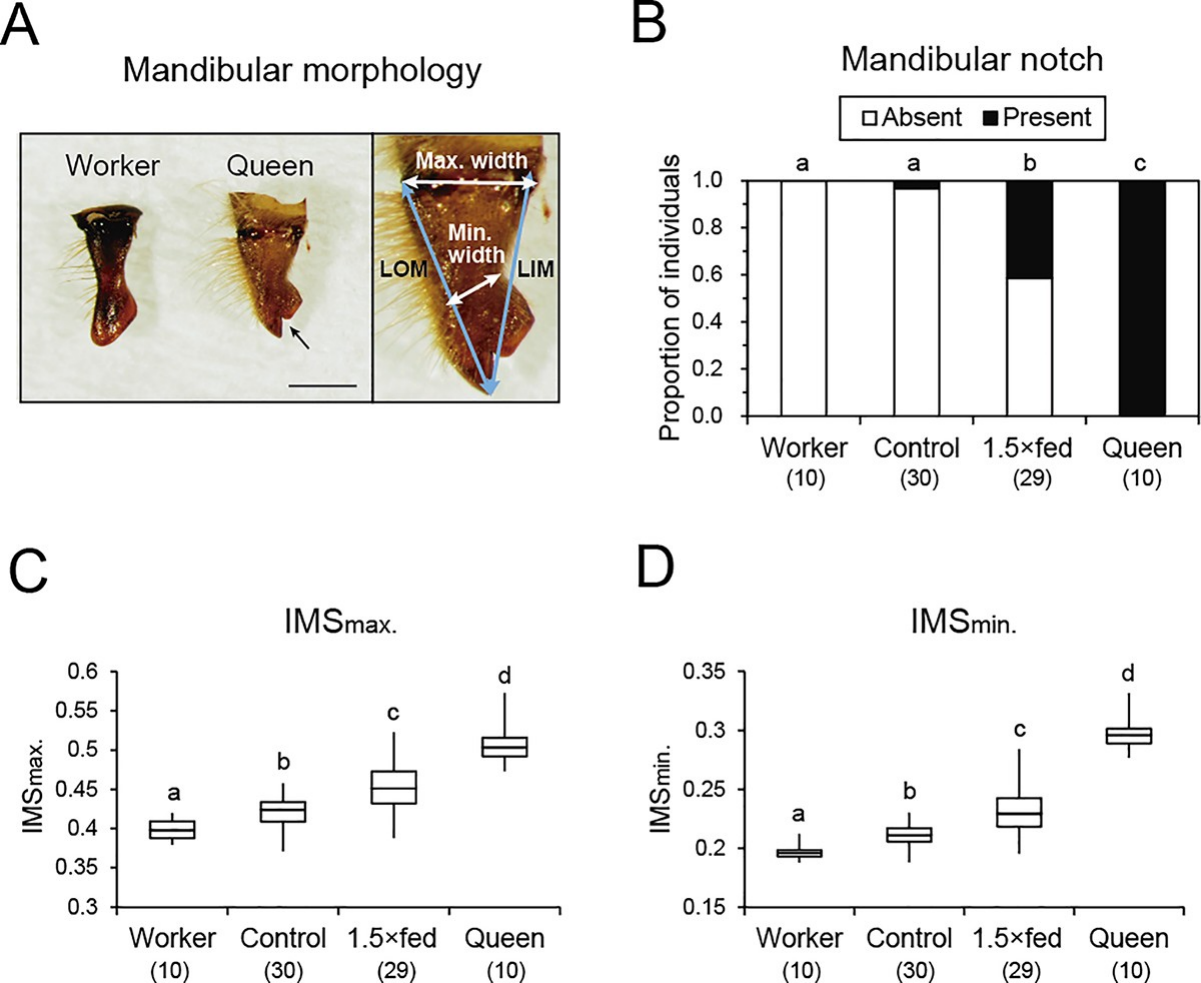
External morphology of females reared naturally and artificially. (A) Mandibular morphology in worker and queen (left), and positions of length measurements (right). The black arrow indicates a mandibular notch. Scale bar indicates 0.6 mm. (B) Proportion of individuals with or without a mandibular notch. Workers and queens were reared naturally in the nest. Control and 1.5×fed females were reared using artificial diet in the laboratory. The values in parentheses indicate the number of samples. (C) IMS_max_ values in workers, control females, 1.5×fed females, and queens. Box plots indicate maximum, upper quartile, median, lower quartile, and minimum values. (D) IMS_min_ values in different groups of females. Different letters above the bars or plots in B, C, and D indicate significant differences (*P* < 0.05).

To evaluate the degree of queen-like morphology, we focused on the mandible as an external morphological characteristic and the reproductive organs as internal morphological characteristics, especially the ovaries and spermatheca, as done previously [3,45]. The length from the tip to the base inside the mandible (LIM) and from the tip to the base outside the mandible (LOM), as well as the maximum width of the mandible base (max. width), and minimum width of the mandible’s middle (min. width), were measured to assess the mandibular morphology (see Results in Fig 1A). To evaluate the proportion of the mandibular length relative to its width, indexes of mandibular shape (IMSs) were calculated as follows:

$$\begin{array}{l} {IMS}_{max.}=\{({max.width}_{right}+{max.width}_{left})÷2\}\\ \;÷\{({LIM}_{right}+{LIM}_{left}+{LOM}_{right}+{LOM}_{left})÷4\} \end{array}$$

$$\begin{array}{l} {IMS}_{min.}=\{({min.width}_{right}+{min.width}_{left})÷2\}\\ \;÷\{({LIM}_{right}+{LIM}_{left}+{LOM}_{right}+{LOM}_{left})÷4\} \end{array}$$

Presence or absence of the mandibular notch was recorded. To evaluate internal morphology of ovaries and spermatheca, the abdomen were dissected with honey bee saline (128.337 mM NaCl, 2.68 mM KCl, 1.8 mM CaCl_2_, pH 6.7) [47] under a dissecting microscope. The number of ovarioles in the ovary were recorded. The spermatheca diameter was also measured twice at different positions and averaged. Regular workers in the honey bee retained a small spermatheca with a measurable spermathecal duct and a muscular pump, and a small spermathecal gland as previously reported [48], whereas artificial reared females possessed these parts and a relatively larger spermathecal reservoir. Therefore, we measured a larger part of spermatheca (a spermathecal duct with a pump for a worker-like female and a spermathecal reservoir for a queen-like female).

### Measurements of dopamine levels in the brain

Frozen brains were dissected in ice-cold saline (pH 6.7) on a Peltier cooling unit (Kenis Ltd, Osaka, Japan) at approximately 4°C under a dissecting microscope. Each dissected brain with a subesophageal ganglion was homogenized for 2 min using a microglass homogenizer in 50 μL ice-cold 0.1 M perchloric acid containing 0.1 ng/μL 3,4-dihydroxyphenylacetic acid. Each sample was then transferred into a 1.5-mL microcentrifuge tube and centrifuged at 15,000 × *g* for 30 min at 4°C. Supernatants were transferred into microvials for analysis by high-performance liquid chromatography with electrochemical detection (HPLC-ECD).

The HPLC-ECD method developed by [21] was used to analyze the levels of dopamine. The HPLC system comprised a solvent delivery pump (PU-2080, JASCO, Tokyo, Japan), a refrigerated automatic injector (AS-2057, JASCO), and a C18 reversed-phase column (250 mm × 4.6 mm internal diameter (id), 5-μm average particle size, UG 120, Osaka Soda, Osaka, Japan) maintained at 35°C. An electrochemical detector (ECD-700, EICOM, Kyoto, Japan) set at 0.85 V was applied at 35°C. The mobile phase comprised 0.18 M monochloroacetic acid and 40 μM 2Na-EDTA, which was adjusted to pH 3.6 with NaOH, and 1.62 mM sodium-1-octanesulfonate and 5% CH_3_CN (v/v%, final concentration) were added to this solution. The flow rate was kept constant at 0.7 mL/min. External standards were run before and after the sample runs to identify and quantify the biogenic amines. Each biogenic amine peak was identified based on comparisons with both the retention times and hydrodynamic voltammograms for those of the standards. Measurements based on the peak areas in the chromatograms were obtained by calculating the ratio of the peak area for a substance relative to the peak area for the standard.

### Evaluation of aggressive behavior

Virgin queen honey bees exhibit high aggressiveness against rival virgin queens [49]. When they encounter other virgin queens, they immediately bite or sting them. To determine whether an intermediate caste female exhibited aggressive behavior against another female of an intermediate caste, two three-day-old females in a single Petri dish (3.5 cm id × 1 cm) were transferred into an arena (8 cm id × 4.5 cm high) with filter paper at the bottom and allowed to adapt for 2 min, after which the two Petri dishes were removed so interactions could occur. A red transparent sheet was placed on the top of the arena to create a darkened environment similar to the colony conditions, as previously reported [17,18]. Interactions between females were observed and recorded using a digital camera. When one female bit and tried to sting another, the fight was interrupted and the aggressive female was removed from the arena. A female that bit or stung another was defined as a winner, whereas a female bitten by or that escaped from another was defined as a loser. When one female encountered another and escaped or did not perform any aggressive behavior for 2 min, they were defined as non-fighting individuals. Adult females were obtained by artificial rearing and fed 1.5 times the amount of control diet during the larval stage. When the females emerged, they were marked by cutting the side of part of a wing and kept in the cage with 10 workers taken from the nest for three days at 32°C in an incubator. After the fighting experiment, the test female was transferred into the cage with 10 workers and kept for one hour, and then euthanized with liquid nitrogen for measurements of dopamine levels and stored until use.

### Measurements of tyrosine, catecholamines, and phenolamines in newly emerged females

Tyrosine is a common potential precursor of catecholamines [Dihydroxyphenylalanine: DOPA (a precursor of dopamine), dopamine, and *N*-acetyldopamine (a metabolite of dopamine)] and phenolamines (tyramine and octopamine) in insects [50,51]. To determine whether tyrosine is converted mainly into catecholamines or phenolamines, the brain levels of tyrosine and those of catecholamines and phenolamines were measured in the brains of test individuals. To exclude the possibility of effects due to behavioral interactions on the biogenic amine levels, newly emerged females were used within 12 h. Newly emerged females fed 1.5 times the amount of control diet were euthanized with liquid nitrogen and stored until use. Frozen brains were removed from the heads and treated according to the same procedures for the quantification of dopamine. We used the HPLC-ECD system described above to quantify the levels of dopamine, *N*-acetyldopamine, tyramine, and octopamine in the brains. In addition, we analyzed the same brain samples using another HPLC-ECD system to quantify the levels of tyrosine and DOPA in the brains.

The HPLC-ECD system for analyzing tyrosine and DOPA developed by [25] comprised a solvent delivery pump (PU-4580, JASCO), a refrigerated automatic injector (AS-4550, JASCO), and a C18 reversed-phase column (250 mm × 4.6 mm id, 5-μm average particle size, MG120, Osaka Soda Ltd, Japan) maintained at 35°C. An electrochemical detector (ECD-300, EICOM) set at 0.8 V was employed at a temperature of 35°C. The mobile phase comprised 83 mM citric acid monohydrate, 17 mM sodium acetate, 13 μM 2Na-EDTA, and 2.3 mM sodium-1-octanesulfonate, and 7% methanol (v/v%, final concentration) was added to this solution. The flow rate was kept constant at 0.7 mL/min. Tyrosine and DOPA were quantified according to the same procedure described above for dopamine.

### Statistical analyses

Parameters comprising IMS_max_, IMS_min_, spermatheca diameter, and the dopamine levels in the brain were compared among regular workers, control females, 1.5×fed females, and regular queens using the Kruskal–Wallis test with the Steel–Dwass multiple comparison test (significant differences were accepted at *P* = 0.05). The proportions of individuals with a mandibular notch and different numbers of ovarioles were compared among the groups using Fisher’s exact test with Holm’s correction.

For the artificially reared females, the dopamine levels were compared between females with or without a mandibular notch using the Mann–Whitney U test. Correlations between the dopamine levels with IMS_max_, IMS_min_, or the spermatheca diameter were examined based on Spearman’s rank correlation coefficients. Associations between brain dopamine levels and differences in the numbers of ovarioles were compared using the Kruskal–Wallis test with the Steel–Dwass multiple comparison test. The dopamine levels in the brains of winners in the fighting experiment were compared with those of losers and non-fighting females using the Steel multiple comparison test (winner vs. loser, winner vs. non-fighting, significant value *P* = 0.05). The correlations between the brain levels of tyrosine and those of catecholamines and phenolamines were examined based on Spearman’s rank correlation coefficients.

## Results

### External morphology

The mandibular morphology was measured in workers, control females, 1.5×fed females, and queens. There were clear caste-specific differences in the morphology of the mandibular notch and the shape of the mandibles (Fig 1A). The mandibular notch was present in all queens but absent from all workers, and the proportions of individuals with the mandibular notch differed significantly among the four groups (Fisher’s exact test, *P* < 0.001, Fig 1B). Individuals with the mandibular notch were observed more frequently in 1.5×fed females (0.414) than control females (0.033) (Holm’s correction, *P* < 0.01). The IMS_max_ values differed significantly among the four groups (Kruskal–Wallis test, H = 45.996, *P* < 0.001), with the largest values in queens and the smallest in workers (Steel–Dwass, *P* < 0.05, Fig 1C). The IMS_max_ values were significantly larger in 1.5×fed times females than control females and workers, but smaller than those in queens. The IMS_max_ values in control females were intermediate between those of workers and 1.5×fed times females. The trends were similar for the IMS_min_ values (Kruskal–Wallis test, H = 50.204, *P* < 0.001; Steel–Dwass, *P* < 0.05, Fig 1D).

### Internal morphology

**Fig 2:**
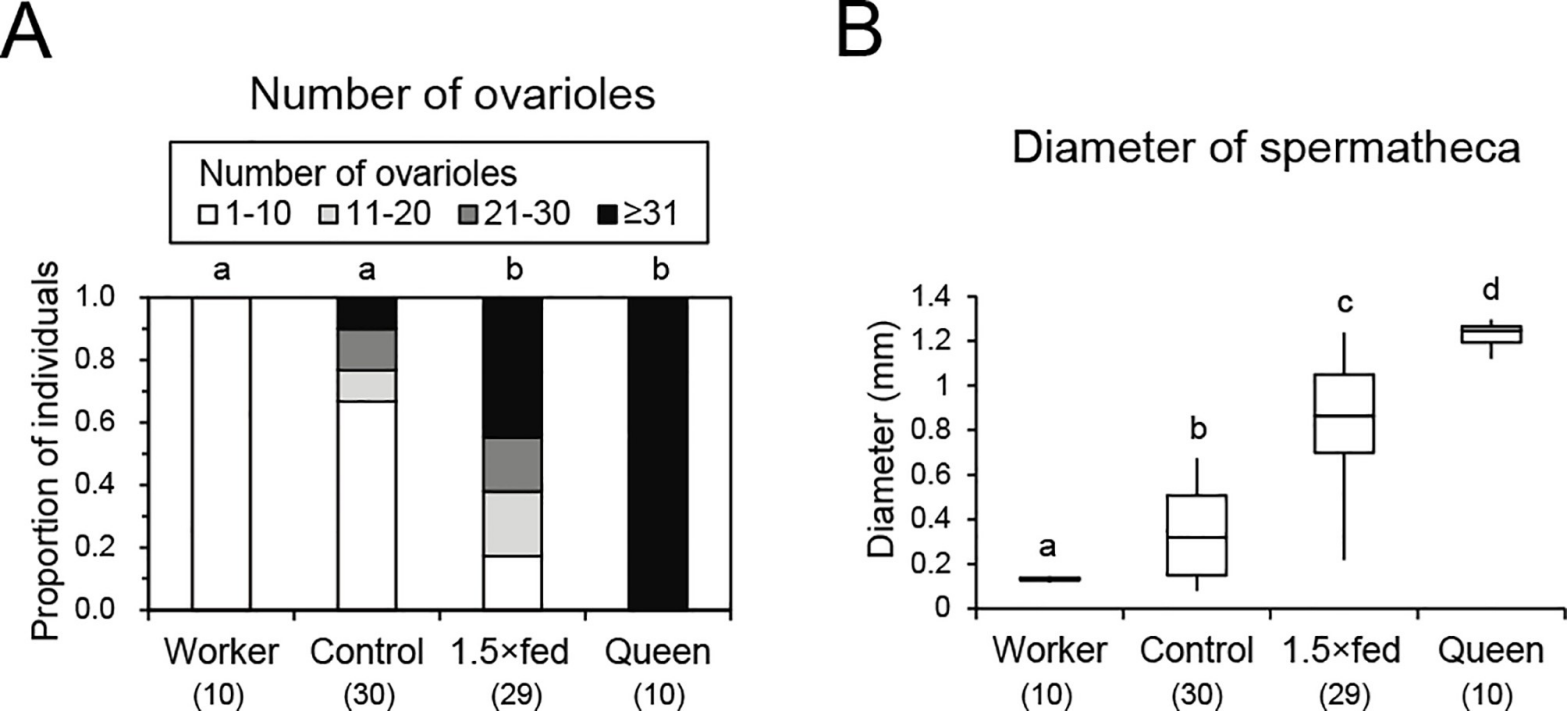
Internal morphology of females reared naturally and artificially. (A) Proportions of individuals with ovaries categorized based on the number of ovarioles. Workers and queens were reared naturally in the nest. Control and 1.5×fed females were reared using artificial diet in the laboratory. The values in parentheses indicate the number of samples. (B) Spermatheca diameters. Different letters above the bars or plots indicate significant differences (*P* < 0.05). Box plots indicate maximum, upper quartile, median, lower quartile, and minimum values.

There were clear caste-specific differences in the morphology of the ovaries and spermatheca. Females reared artificially possessed intermediate characteristics between regular workers and queens reared naturally. The number of ovarioles differed significantly among workers, control females, 1.5×fed females, and queens (Fisher’s exact test, *P* < 0.001, Fig 2A). All queens had more than 31 ovarioles, whereas all workers had 1–10 ovarioles. The number of ovarioles in 1.5×fed females was significantly larger than those in control females and workers (Holm’s correction, *P* < 0.01), but it differed only marginally from that in queens (*P* = 0.06). The number of ovarioles in control females did not differ significantly from that in workers (*P* = 0.351) but it was lower than those in 1.5×fed females and queens (*P* < 0.001). The spermatheca diameter differed significantly among workers, control females, 1.5×fed females, and queens (Kruskal–Wallis test, H = 57.651, *P* < 0.001), where the diameter was largest in queens but smallest in workers (Steel–Dwass, *P* < 0.05, Fig 2B). The spermatheca diameter was significantly larger in 1.5×fed females than control females and workers but smaller than that in queens (*P* < 0.05). The spermatheca diameter was significantly larger in control females than workers but smaller than those in 1.5×fed females and queens (*P* < 0.05).

### Brain levels of dopamine

**Fig 3:**
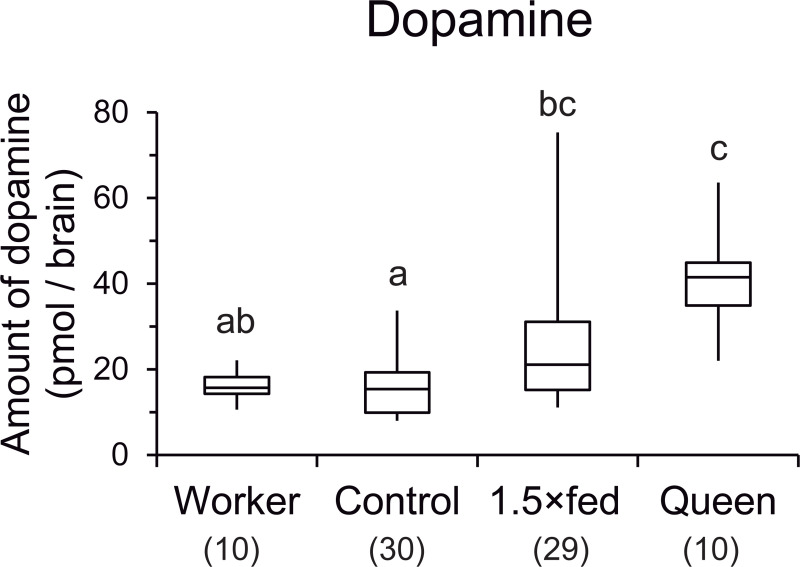
Dopamine levels in the brain among females reared naturally and artificially. Workers and queens were reared naturally in the nest. Control and 1.5×fed females were reared using artificial diet in the laboratory. The values in parentheses indicate the number of samples. Different letters above the plots indicate significant differences (*P* < 0.05). Box plots indicate maximum, upper quartile, median, lower quartile, and minimum values.

There were clear caste differences in dopamine production as previously reported [15,16]. Females reared artificially had intermediate levels of dopamine between regular workers and queens reared naturally. The amounts of dopamine in the brain differed significantly among workers, control females, 1.5×fed females, and queens (Kruskal–Wallis test, H = 28.009, *P* < 0.001, Fig 3). The dopamine levels differed significantly between workers and queens (Steel–Dwass, *P* < 0.05). The dopamine level was significantly higher in 1.5×fed females than control females (*P* < 0.05), and intermediate between those in workers and queens. The dopamine level was significantly lower in control females than queens (*P* < 0.05), but not different from that in workers.

### Correlations between dopamine levels and morphological characteristics

**Fig 4:**
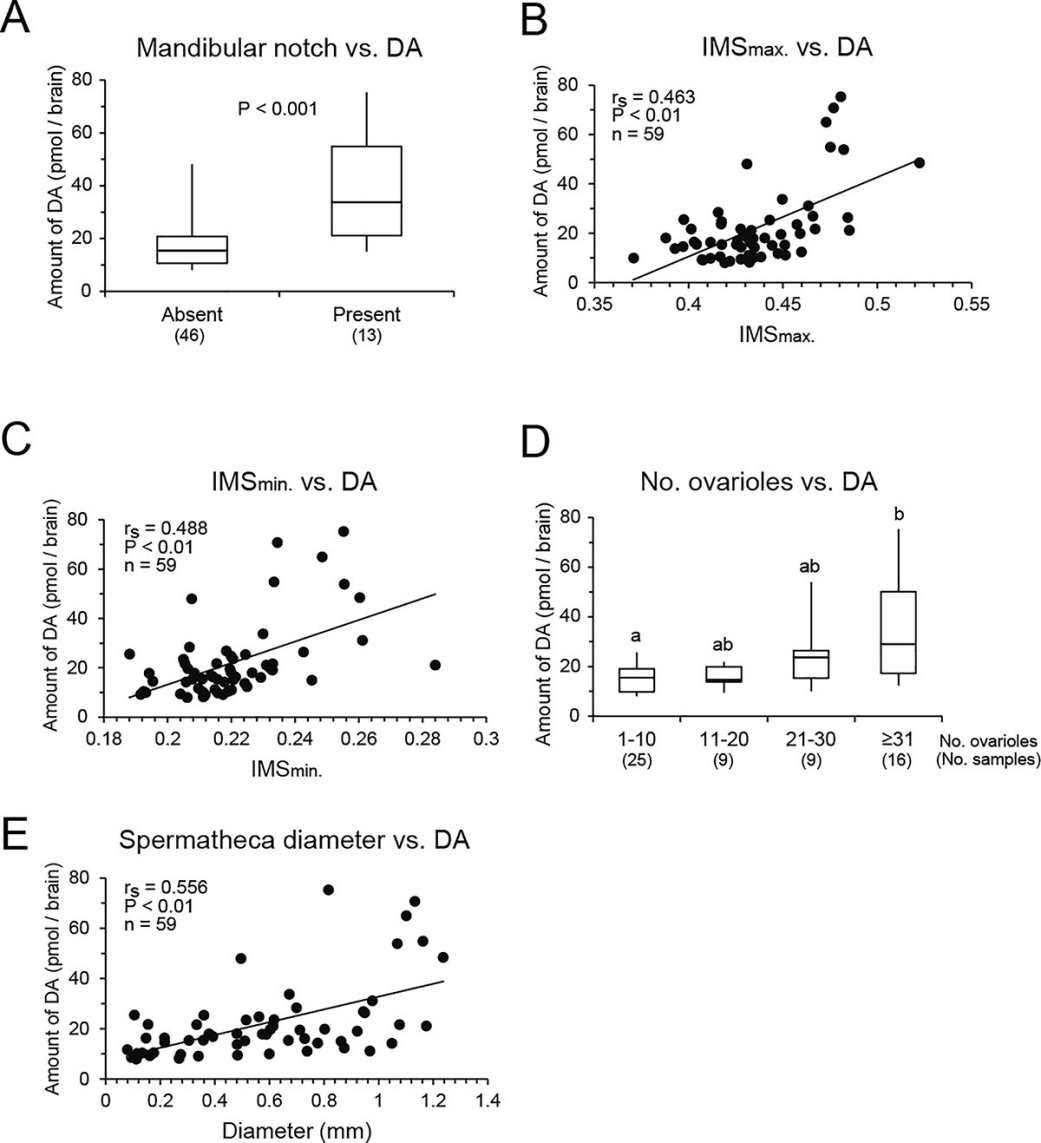
Relationships between brain dopamine levels and morphological parameters in artificially reared females. (A) Amounts of dopamine (DA) in females with or without a mandibular notch. (B) Correlation between IMS_max_ and DA level in the brain. (C) Correlation between IMS_min_ and DA level in the brain. (D) Amounts of DA in females with ovaries categorized based on the number of ovarioles. Different letters above the plots indicate significant differences (*P* < 0.05). (E) Correlation between spermatheca diameter and DA level in the brain. The values in parentheses in A and D indicate the number of samples. Box plots indicate maximum, upper quartile, median, lower quartile, and minimum values.

Examinations of the external morphology of artificially reared females showed that all but one of the control females lacked a mandibular notch, whereas 12/29 of the 1.5×fed females had a mandibular notch (Figs 1B and 4A). The dopamine level was significantly higher in females with a mandibular notch than those without a mandibular notch (Mann–Whitney U test, z = 4.078, *P* < 0.001, Fig 4A). The IMS_max_ values were positively correlated with the dopamine levels in the brain (Spearman’s rank correlation, r_s_ = 0.463, *P* < 0.01, n = 59, Fig 4B). The IMS_min_ values were also positively correlated with the dopamine levels in the brain (r_s_ = 0.488, *P* < 0.01, n = 59, Fig 4C). Examinations of the internal morphology showed that the dopamine level in the brain differed significantly among the four groups with different numbers of ovarioles (Kruskal–Wallis test, H = 14.066, *P* < 0.01, Fig 4D). Females with more than 31 ovarioles had the highest amounts of dopamine in the brain, whereas females with 1–10 ovarioles had the lowest (Steel–Dwass, *P* < 0.05, Fig 4D). Females with 11–20 and 21–30 ovarioles had intermediate amounts of dopamine in the brain. The spermatheca diameter was positively correlated with the dopamine level in the brain (Spearman’s rank correlation, r_s_ = 0.556, *P* < 0.01, n = 59, Fig 4E).

### Fighting behavior and dopamine levels in the brain

**Fig 5:**
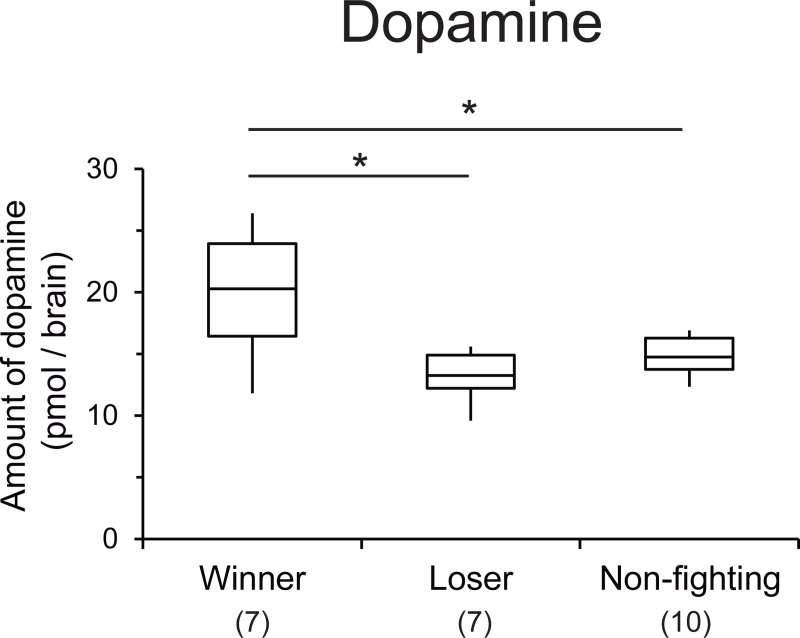
Dopamine levels in the brains of winners, losers, and non-fighting females. Box plots indicate maximum, upper quartile, median, lower quartile, and minimum values. The values in parentheses indicate the number of samples. Asterisks indicate significant differences between groups (*P* < 0.05).

Fighting behavior was observed between artificially reared three-day-old 1.5×fed females. After entering the arena, some females initiated fighting behavior, including biting and stinging, whereas others exhibited no aggressive or fighting behavior and they usually escaped from the female they met in the arena. We categorized females as winners, losers, and non-fighting females. The levels of dopamine in the brain were significantly higher in winners than losers and non-fighting females (Steel test, *P* < 0.05, Fig 5).

### Correlations of brain levels of tyrosine with those of catecholamines and phenolamines

**Fig 6:**
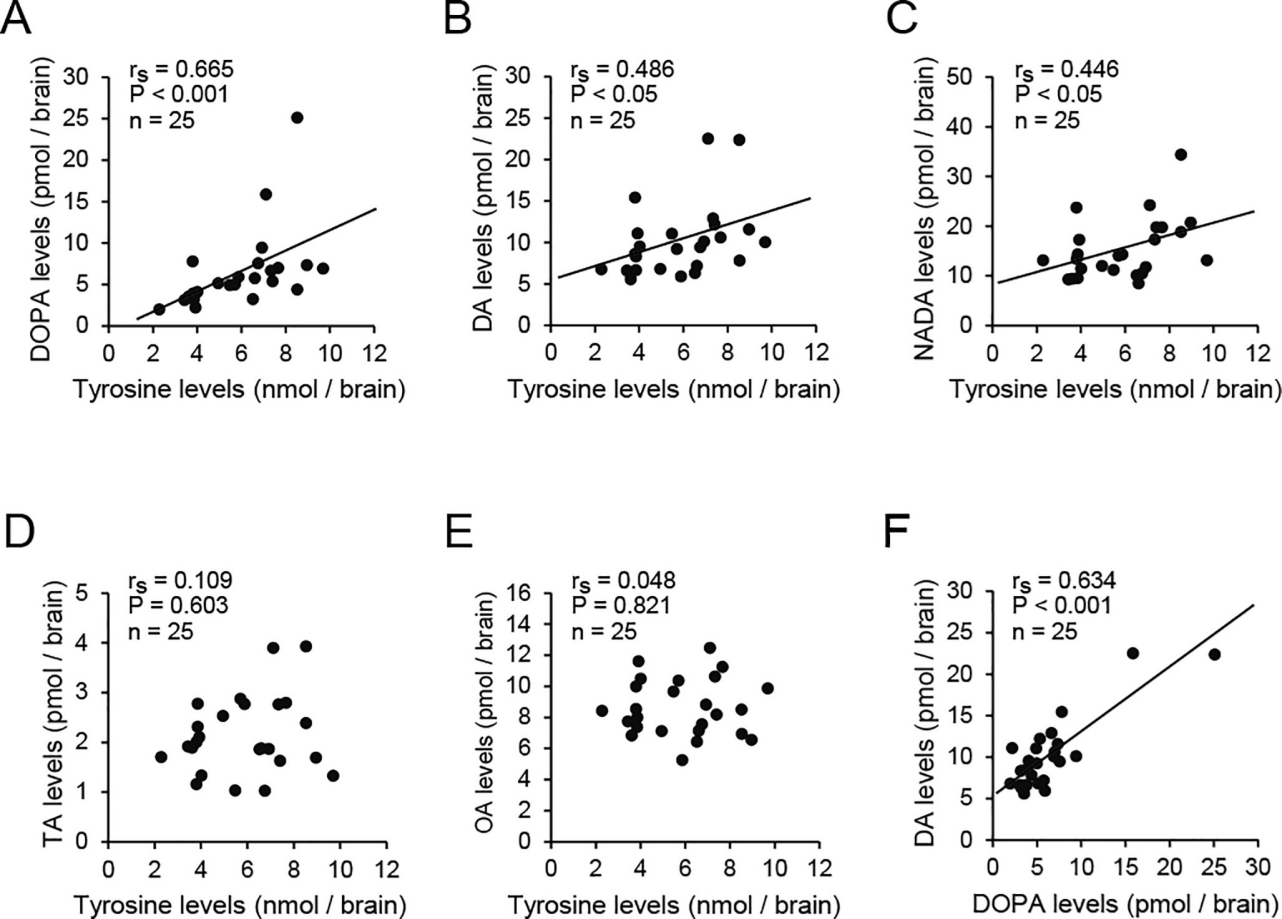
Correlations among the levels of biogenic amines in the brain. Tyrosine levels were positively correlated with the DOPA levels (A), dopamine (DA) levels (B), and *N*-acetyldopamine (NADA) levels (C), but not significantly correlated with the tyramine (TA) levels (D) and octopamine (OA) levels (E). Positive correlations between the DOPA and DA levels are also indicated (F).

The brain levels of tyrosine were positively correlated with those of DOPA, dopamine, and *N*-acetyldopamine in newly emerged 1.5×fed females (Spearman’s rank correlation, DOPA: r_s_ = 0.665, *P* < 0.001, n = 25, Fig 6A; dopamine: r_s_ = 0.486, *P* < 0.05, n = 25, Fig 6B; *N*-acetyldopamine: r_s_ = 0.446, *P* < 0.05, n = 25, Fig 6C). However, the levels of tyrosine were not significantly correlated with those of tyramine and octopamine (tyramine: r_s_ = 0.109, P = 0.603, n = 25, Fig 6D; octopamine: r_s_ = 0.048, *P* = 0.821, n = 25, Fig 6E). The brain levels of DOPA were positively correlated with those of dopamine (r_s_ = 0.634, *P* < 0.001, n = 25, Fig 6F).

## Discussion

In the present study, we successfully obtained females intermediate between workers and queens by artificial rearing, and demonstrated their continuous caste-specific phenotypic characteristics in terms of the morphology, dopamine levels, and aggressive behavior in this advanced eusocial bee. Previous studies reported the production of intermediate external and internal morphological characteristics after treatment with sugar-rich diet [44,52] or juvenile hormone [3,6,10,52]. To our knowledge, however, there have been no reports of intermediate characteristics in terms of the brain dopamine levels and aggressive behavior. Our results highlight the plasticity of the brain substances corresponding to the continuous functional morphology, thereby supporting adaptive behavior based on the morphology.

In the honey bee, caste is exhibited in terms of discontinuous characteristics that are specialized for each task under natural conditions. Our study, based on artificial rearing, showed that feeding larvae with 1.5 times the amount of control diet could generate highly variable parameters in terms of the mandibular morphology, reproductive organs, dopamine level in the brain, and aggressive behavior in adult honey bees, thereby suggesting that these caste-specific characteristics are potentially continuous. If workers feed larger amounts of diet to female larvae under natural conditions, they might develop into females with characteristics intermediate between workers and queens. However, intermediate individuals are found rarely in natural colonies [2,3], and thus the amount of food provided to larvae might be accurately controlled by nurse bees. This accurate control of the amount of food might be applied in advanced eusocial bees to allow the differentiation of discontinuous phenotypic characteristics [10]. The artificially produced continuous characteristics in the caste-specific honey bee phenotype are similar to those found in primitively eusocial bees, including bumble bees, where the morphological characteristics partly overlap between castes. However, little is known about a relationship between nutrition and the continuous characteristics in primitively eusocial species. Investigations of morphological plasticity with nutritional manipulation during larval stage in these species are required.

The caste-specific characteristics investigated in the present study were positively correlated with the dopamine level in the brains of three-day-old females. In the honey bee, the mandibular morphology, number of ovarioles, and spermatheca diameter can be determined from the late larval to prepupal stage [33–35], whereas caste-specific differences in the dopamine level in the brain appear in the late pupal stage [16,36,37]. Therefore, dopamine production may be controlled base on the external and internal morphology, and dopamine might act on the peripheral organs to affect the activities of the ovary and spermatheca.

A previous study of the fighting behavior of virgin queens found that a dopamine receptor antagonist could suppress the aggressive behavior of virgin queens against rival virgin queens depending on its concentration [17], and also suppress the locomotor activities [18]. However, the association between the dopamine levels and degree of aggressiveness in virgin queens was not investigated. In the present study, we artificially produced intermediate individuals with differences in their dopamine and aggressiveness levels, and determined whether the relationship between the brain dopamine level and aggression level was positive or not. Our results showed that winners had higher dopamine levels in the brain than the losers, thereby supporting our prediction of a relationship between dopamine and aggression in virgin queens. A previous study of the ant *Harpegnathos saltator* found that aggressive females had significantly higher dopamine levels at the start of a tournament to determine dominance of the reproductive hierarchy, and females policed by their nestmates also had lower levels of dopamine in the brain [31]. Similar suppression of dopamine in the brain after receiving dominance interactions was reported in the ant *Diacamma* sp. [53]. In the fruit fly, two pairs of dopaminergic neurons that project into the central complex of the brain modulate aggression, but they have no major effects on other behaviors [54]. In the honey bee, there are large caste-specific differences in the dopamine levels in the brain, but no additional clusters of dopaminergic neurons in the brains of queens [16]. Therefore, the caste-specific differences in dopamine levels may be caused by upregulated dopamine synthesis in particular dopaminergic neurons shared with workers. Several dopaminergic neurons shared with workers project into the central complex in the brain [55–57]. The clusters of dopaminergic neurons involved in fighting behavior need to be determined in future research.

The brain dopamine levels in the intermediate caste females at emergence might have been influenced greatly by the amount of the dopamine precursor tyrosine in the brain. Our results showed that the brain levels of tyrosine were positively correlated with the levels of DOPA, dopamine, and *N*-acetyldopamine, but not significantly correlated with the levels of the phenolamines that we examined. These results suggest a strong metabolic relationship where the tyrosine in the brain is mainly converted into catecholamines. This might be partly due to the activities of enzymes involved in dopamine biosynthesis, because newly emerged queens showed higher expression levels of enzyme genes (tyrosine hydroxylase and DOPA decarboxylase) in the brains than workers [16]. However, the process of tyrosine supply in the brains is still unclear. The source of tyrosine might have been the artificial diet, especially royal jelly in the larval stage, because tyrosine is present in royal jelly as one of the 26 amino acids it is comprised of [58–60], although tyrosine is not the most abundant amino acid in royal jelly. It has also been reported that the amounts of tyrosine in the hemolymph are higher in queens than workers [61]. The intake of royal jelly or tyrosine can elevate the brain dopamine level during the adult stage in both female and male honey bees [25,62], and this might also apply to the intake of royal jelly during the larval stage. Thus, female larvae given 1.5 times the amounts of control diet might have consumed larger amounts of tyrosine during the larval stage, which was converted into more DOPA, dopamine, and *N*-acetyldopamine during the pupal stage. Thus, the larval nutritional state may be linked to the intake of tyrosine to influence the brain level of dopamine at emergence and caste-specific behavior including aggression during the early adult stage in honey bee females.

## Conclusion

In this study, we determined whether caste-specific discontinuous characteristics of the external and internal morphology, brain dopamine level, and aggressive behavior can be made continuous by artificially rearing with different amounts of diet. The morphological characteristics of the mandible and reproductive organs, as well as the levels of dopamine in the brain, were discontinuous between workers and queens reared naturally in the nest. However, females given 1.5 times the amount of control diet possessed morphological characteristics intermediate between castes. These females also had intermediate levels of dopamine in the brain. In fighting experiments, the winners had higher dopamine levels in the brain than the losers. The brain levels of tyrosine were positively correlated with those of DOPA, dopamine, and *N*-acetyldopamine, thereby suggesting a strong metabolic relationship between tyrosine and dopamine in the brain. Thus, caste-specific characteristics are potentially continuous depending on the amount of food provided, and this property may be conserved among primitive and advanced eusocial bees. The dopamine level in the brain is also a caste-specific characteristic linked to the nutritional state during the larval stage and it may be associated with aggressive behavior in queen-type females.

## Supporting information

S1 Table Daily amounts and concentration of artificial diet fed to larvae. (PDF)

S2 Table Individual data of mandibular morphology (mandibular notch, IMSmax. and IMSmin.), number of ovarioles, spermatheca diameter and brain levels of dopamine in females in honey bees. (PDF)

S3 Table Brain levels of dopamine in 1.5×fed females for fighting experiments. (PDF)

S4 Table Monoamine levels in the brains in 1.5×fed females at emergence. (PDF)
