# Supplementary material for: Dopamine production in the brain is associated with caste-specific morphology and behavior in an artificial intermediate honey bee caste
Source: PLoS One. 2020 Dec 17;15(12):e0244140. doi: 10.1371/journal.pone.0244140 (PMC7746283; doi:10.1371/journal.pone.0244140)
Supplement: S1 Table — (PDF) [file pone.0244140.s001.pdf]

S1 Table. Daily amounts and concentration of artificial diet fed to larvae.

Larval day age	Composition (w/w%)*					Amount (μL)	
	Glucose	Fructose	Yeast	Royal jelly	Water	Control fed condition	1.5 times fed condition
1	6	6	1		37	10	15
2	6	6	1		37	10	15
3	7.5	7.5	1.5	50	33.5	20	30
4	9	9	2		30	30	45
5	9	9	2		30	40	60
6	9	9	2		30	50	75

* Glucose, fructose and yeast were dissolved in sterile water, and then mixed with the same weight of liquid royal jelly.
