# Supplementary material for: Dopamine production in the brain is associated with caste-specific morphology and behavior in an artificial intermediate honey bee caste
Source: PLoS One. 2020 Dec 17;15(12):e0244140. doi: 10.1371/journal.pone.0244140 (PMC7746283; doi:10.1371/journal.pone.0244140)
Supplement: S2 Table — (PDF) [file pone.0244140.s002.pdf]

S2 Table. Individual data of mandibular morphology (mandibular notch, IMSmax. and IMSmin.), number of ovarioles, spermatheca diameter and brain levels of dopamine in females in honey bees.

Id.	Group	Food	Mandibular notch	IMSmax.	IMSmin.	No. ovarioles	Spermatheca diameter	Dopamine (pmol/brain)
NW-01	Worker	-	Absent	0.3957	0.19292	4	0.141	17.0921688
NW-02	Worker	-	Absent	0.41954	0.19181	4	0.13	14.0546588
NW-03	Worker	-	Absent	0.39022	0.18765	5	0.1175	18.6214779
NW-04	Worker	-	Absent	0.37892	0.19868	2	0.1265	15.2150834
NW-05	Worker	-	Absent	0.38295	0.20101	4	0.135	22.0675543
NW-06	Worker	-	Absent	0.38704	0.19726	3	0.1325	14.7704137
NW-07	Worker	-	Absent	0.41223	0.21202	4	0.116	16.2458901
NW-08	Worker	-	Absent	0.40028	0.19681	6	0.14	19.7005159
NW-09	Worker	-	Absent	0.41213	0.1936	6	0.144	14.2058358
NW-10	Worker	-	Absent	0.40023	0.19492	4	0.135	10.6535649
CF01-01	Control	Control	Absent	0.43136	0.21736	9	0.118	9.20634667
CF01-02	Control	Control	Absent	0.40764	0.2118	9	0.34	9.13415156
CF01-03	Control	Control	Absent	0.41903	0.20612	4	0.113	7.9891567
CF01-04	Control	Control	Absent	0.43463	0.21104	10	0.116	10.2177316
CF01-05	Control	Control	Absent	0.44729	0.20964	5	0.08	11.7713965
CF02-01	Control	Control	Absent	0.4275	0.21537	16	0.156	21.7400833
CF02-02	Control	Control	Absent	0.45732	0.20495	3	0.515	23.5206979
CF02-03	Control	Control	Absent	0.40707	0.1915	4	0.162	9.30882913
CF02-04	Control	Control	Absent	0.43825	0.21897	4	0.134	10.4156315
CF02-05	Control	Control	Absent	0.42528	0.20772	25	0.67	15.3777292
CF03-01	Control	Control	Absent	0.39739	0.18807	9	0.105	25.5622952
CF03-02	Control	Control	Absent	0.43151	0.19421	31 <	0.574	17.8763369
CF03-03	Control	Control	Absent	0.40134	0.20543	3	0.334	21.6676307
CF04-01	Control	Control	Absent	0.43216	0.21114	10	0.269	8.2271468
CF04-02	Control	Control	Absent	0.44024	0.21987	26	0.481	18.0917995
CF04-03	Control	Control	Absent	0.4115	0.21575	9	0.275	9.82305481
CF04-04	Control	Control	Absent	0.3927	0.22419	16	0.482	13.7642939
CF04-05	Control	Control	Absent	0.42177	0.2115	4	0.095	8.63652329
CF05-01	Control	Control	Absent	0.37072	0.19315	26	0.6	9.9896153
CF05-02	Control	Control	Absent	0.42902	0.20633	9	0.603	19.6648199
CF05-03	Control	Control	Absent	0.41662	0.19242	6	0.176	10.4959067
CF05-04	Control	Control	Absent	0.4277	0.20402	14	0.484	9.46279853
CF05-05	Control	Control	Absent	0.43416	0.20839	31 <	0.589	17.8610789
CF06-01	Control	Control	Absent	0.41718	0.22046	29	0.617	23.678361
CF06-02	Control	Control	Absent	0.40278	0.21413	10	0.216	16.3925188
CF06-03	Control	Control	Absent	0.4115	0.22133	5	0.148	16.299223
CF06-04	Control	Control	Absent	0.44287	0.22443	2	0.36	25.3999512
CF06-05	Control	Control	Absent	0.4041	0.21085	5	0.358	15.5115305
CF06-06	Control	Control	Absent	0.41754	0.21557	7	0.306	15.3953803
CF06-07	Control	Control	Present	0.4496	0.22993	31 <	0.673	33.7621395
1.5F01-01	1.5×fed	1.5×fed	Absent	0.43185	0.22001	11	0.738	11.0687192
1.5F01-02	1.5×fed	1.5×fed	Present	0.45902	0.23216	20	0.803	19.8663958
1.5F01-03	1.5×fed	1.5×fed	Absent	0.43282	0.22919	9	0.729	16.1530844
1.5F01-04	1.5×fed	1.5×fed	Absent	0.45093	0.22059	31 <	0.51	15.2204281
1.5F01-05	1.5×fed	1.5×fed	Absent	0.44882	0.21956	19	0.712	19.5067269
1.5F02-01	1.5×fed	1.5×fed	Absent	0.46587	0.21837	31 <	0.944	26.863137
1.5F02-02	1.5×fed	1.5×fed	Absent	0.45968	0.2249	31 <	0.874	12.3936162
1.5F02-03	1.5×fed	1.5×fed	Present	0.46698	0.23296	20	1.077	21.6935713
1.5F02-04	1.5×fed	1.5×fed	Absent	0.43486	0.21779	31 <	1.049	14.2461408
1.5F02-05	1.5×fed	1.5×fed	Present	0.46348	0.26108	31 <	0.977	31.1251065
1.5F03-01	1.5×fed	1.5×fed	Present	0.47507	0.23337	31 <	1.163	54.8917437
1.5F03-02	1.5×fed	1.5×fed	Present	0.47684	0.23444	31 <	1.133	70.7910369
1.5F03-03	1.5×fed	1.5×fed	Present	0.44427	0.2452	31 <	0.863	15.0166182

1.5F04-01	1.5×fed	1.5×fed	Absent	0.41734	0.21985	22	0.562	24.7619951
1.5F04-02	1.5×fed	1.5×fed	Absent	0.42586	0.21353	7	0.393	16.9221053
1.5F04-03	1.5×fed	1.5×fed	Present	0.43246	0.23286	8	0.922	19.0921308
1.5F04-04	1.5×fed	1.5×fed	Absent	0.45129	0.21477	21	0.968	11.1574901
1.5F05-01	1.5×fed	1.5×fed	Present	0.48519	0.28396	31 <	1.175	21.1190083
1.5F05-02	1.5×fed	1.5×fed	Present	0.5223	0.26029	31 <	1.237	48.4904672
1.5F05-03	1.5×fed	1.5×fed	Absent	0.48428	0.24263	28	0.949	26.3849991
1.5F05-04	1.5×fed	1.5×fed	Absent	0.42777	0.20575	11	0.777	14.311441
1.5F05-05	1.5×fed	1.5×fed	Present	0.47282	0.24844	31 <	1.102	64.9822578
1.5F06-01	1.5×fed	1.5×fed	Absent	0.41558	0.20679	21	0.7	28.4132819
1.5F06-02	1.5×fed	1.5×fed	Absent	0.39696	0.19535	17	0.217	14.5763805
1.5F06-03	1.5×fed	1.5×fed	Absent	0.38778	0.2265	4	0.379	18.0308315
1.5F06-04	1.5×fed	1.5×fed	Present	0.48055	0.25516	31 <	0.817	75.287871
1.5F06-05	1.5×fed	1.5×fed	Absent	0.4309	0.20753	31 <	0.496	48.0411003
1.5F06-06	1.5×fed	1.5×fed	Absent	0.43311	0.231	9	0.614	21.1468502
1.5F06-07	1.5×fed	1.5×fed	Present	0.48217	0.2554	26	1.069	53.9287571
NQ-01	Queen	-	Present	0.50943	0.30245	31 <	1.119	34.5868115
NQ-02	Queen	-	Present	0.51774	0.29657	31 <	1.2945	46.1665234
NQ-03	Queen	-	Present	0.48914	0.28759	31 <	1.2355	35.8316128
NQ-04	Queen	-	Present	0.50034	0.29293	31 <	1.1915	39.4194807
NQ-05	Queen	-	Present	0.47281	0.27669	31 <	1.145	45.3913182
NQ-06	Queen	-	Present	0.48095	0.29543	31 <	1.264	43.5215628
NQ-07	Queen	-	Present	0.50547	0.29736	31 <	1.1935	43.6334026
NQ-08	Queen	-	Present	0.50105	0.2837	31 <	1.254	21.9883769
NQ-09	Queen	-	Present	0.57252	0.32451	31 <	1.294	33.8595991
NQ-10	Queen	-	Present	0.53466	0.33151	31 <	1.265	63.6423379
