# Supplementary material for: Dopamine production in the brain is associated with caste-specific morphology and behavior in an artificial intermediate honey bee caste
Source: PLoS One. 2020 Dec 17;15(12):e0244140. doi: 10.1371/journal.pone.0244140 (PMC7746283; doi:10.1371/journal.pone.0244140)
Supplement: S3 Table — (PDF) [file pone.0244140.s003.pdf]

S3 Table. Brain levels of dopamine in 1.5×fed females for fighting experiments.

Pair	Behavioral state	Dopamine (pmol/brain)
P02	Winner	26.00562551
P03	Winner	11.52545659
P06	Winner	22.82830259
P08	Winner	15.62633081
P09	Winner	17.87427451
P10	Winner	21.28894985
P11	Winner	26.07261746
	mean	20.17450819
P02	Loser	11.67415153
P03	Loser	9.442128585
P06	Loser	12.62703637
P08	Loser	15.79669128
P09	Loser	15.21162864
P10	Loser	15.33583567
P11	Loser	13.34791201
	mean	13.34791201
P01	Non-fighting	15.89357026
P01	Non-fighting	14.14360672
P04	Non-fighting	14.37944009
P04	Non-fighting	12.27932394
P05	Non-fighting	16.09582736
P05	Non-fighting	14.37944009
P07	Non-fighting	13.75983167
P07	Non-fighting	13.26775463
P12	Non-fighting	17.05708725
P12	Non-fighting	16.44894789
	mean	14.77048299
