# Supplementary material for: Dopamine production in the brain is associated with caste-specific morphology and behavior in an artificial intermediate honey bee caste
Source: PLoS One. 2020 Dec 17;15(12):e0244140. doi: 10.1371/journal.pone.0244140 (PMC7746283; doi:10.1371/journal.pone.0244140)
Supplement: S4 Table — (PDF) [file pone.0244140.s004.pdf]

S4 Table. Monoamine levels in the brains in 1.5×fed females at emergence.

| ID. | Group | Tyrosine (nmol/brain) | DOPA (pmol/brain) | Dopamine (pmol/brain) | NADA (pmol/brain) | Tyramine (pmol/brain) | Octopamine (pmol/brain) |
|---|---|---|---|---|---|---|---|
| 1.5F09-01 | 1.5×fed | 6.93282152 | 9.41218688 | 10.0846673 | 11.7593971 | 1.86509309 | 8.82543133 |
| 1.5F09-02 | 1.5×fed | 3.80626246 | 3.91568409 | 8.59649858 | 13.4043677 | 2.00569063 | 8.53278608 |
| 1.5F09-03 | 1.5×fed | 3.85792437 | 3.193822 | 8.31131795 | 14.3385617 | 2.77548088 | 7.99666203 |
| 1.5F09-04 | 1.5×fed | 4.95455558 | 5.13985144 | 6.78319297 | 12.0142443 | 2.52761922 | 7.10406039 |
| 1.5F09-05 | 1.5×fed | 3.92452531 | 2.20380673 | 11.0635787 | 17.2605288 | 2.10287407 | 11.5993586 |
| 1.5F09-06 | 1.5×fed | 5.4801932 | 4.92235556 | 11.0367957 | 11.2376059 | 1.03079077 | 9.65577425 |
| 1.5F09-07 | 1.5×fed | 7.4156343 | 5.34915568 | 12.1764358 | 19.797506 | 1.63033166 | 8.18351657 |
| 1.5F09-08 | 1.5×fed | 9.70201792 | 6.90507732 | 10.0449606 | 13.1407739 | 1.32948802 | 9.85120861 |
| 1.5F09-09 | 1.5×fed | 7.6734462 | 6.99119941 | 10.5744624 | 19.8231882 | 2.7955123 | 11.235451 |
| 1.5F09-10 | 1.5×fed | 3.61601879 | 3.5269431 | 5.5591954 | 9.43581914 | 1.8918208 | 6.82253437 |
| 1.5F09-11 | 1.5×fed | 5.87998835 | 5.89266438 | 5.90892624 | 14.3375313 | 2.76718006 | 5.24964057 |
| 1.5F09-12 | 1.5×fed | 8.535606 | 4.39644561 | 7.80927734 | 18.8495507 | 3.92438144 | 6.93328264 |
| 1.5F09-13 | 1.5×fed | 8.96638867 | 7.32824655 | 11.5678813 | 20.7331719 | 1.69285288 | 6.54601019 |
| 1.5F09-14 | 1.5×fed | 7.34554024 | 6.64479725 | 12.872933 | 17.3594164 | 2.76128154 | 10.6192758 |
| 1.5F09-15 | 1.5×fed | 6.75774273 | 7.55672704 | 9.44257436 | 10.5444157 | 1.02806302 | 7.54557922 |
| 1.5F10-01 | 1.5×fed | 3.855027 | 3.83746485 | 6.63893565 | 9.58001525 | 2.31117585 | 7.366618 |
| 1.5F10-02 | 1.5×fed | 3.43737171 | 3.09407388 | 6.59092455 | 9.34867355 | 1.91242846 | 7.73963784 |
| 1.5F10-03 | 1.5×fed | 5.70109464 | 4.97200299 | 9.21265366 | 14.0735108 | 2.87603959 | 10.3672549 |
| 1.5F10-04 | 1.5×fed | 4.01618183 | 4.07934608 | 9.50726658 | 11.4956083 | 1.33131596 | 10.4861312 |
| 1.5F10-05 | 1.5×fed | 6.60984563 | 5.74381309 | 7.1651882 | 8.50996027 | 1.87907817 | 7.13576344 |
| 1.5F10-06 | 1.5×fed | 3.80381498 | 7.79011124 | 15.4101917 | 23.7480245 | 1.1580936 | 9.98214507 |
| 1.5F10-07 | 1.5×fed | 8.52963306 | 25.11298 | 22.3399606 | 34.3752665 | 2.38268948 | 8.4997247 |
| 1.5F10-08 | 1.5×fed | 7.11330109 | 15.8430133 | 22.4958659 | 24.2289836 | 3.89304261 | 12.4665307 |
| 1.5F10-09 | 1.5×fed | 2.28391162 | 1.9868815 | 6.76682154 | 13.1706505 | 1.7040466 | 8.42065485 |
| 1.5F10-10 | 1.5×fed | 6.52725028 | 3.22110098 | 6.29988126 | 10.1285757 | 1.85804657 | 6.42454611 |
